# EDESIA: Plants, Food and Health: A cross‐disciplinary PhD programme from crop to clinic

**DOI:** 10.1111/nbu.12565

**Published:** 2022-06-16

**Authors:** Ian M. Clark, Aedin Cassidy, Audrey Heppleston, Mark Bal, Yvie Morgan, Alicia Nicklin, Yang Yue, Aryana Zardkoohi, Cathie Martin

**Affiliations:** ^1^ School of Biological Sciences University of East Anglia Norwich UK; ^2^ School of Biological Sciences, Institute of Global Food Security Queen's University Belfast Belfast UK; ^3^ Genes in the Environment John Innes Centre Norwich UK; ^4^ Molecules from Nature John Innes Centre Norwich UK; ^5^ Gut Microbes and Health Quadram Institute Bioscience Norwich UK

**Keywords:** ageing, diet, health, fruit and vegetables, plant foods

## Abstract

In an era where preventive medicine is increasingly important due to an ageing population and rising obesity, optimised diets are key to improving health and reducing risk of ill health. The Wellcome Trust‐funded, EDESIA: Plants, Food and Health: a cross‐disciplinary PhD programme from Crop to Clinic (218 467/Z/19/Z) focuses on investigating plant‐based nutrition and health, from crop to clinic, drawing on the world‐class interdisciplinary research expertise of partner institutions based on the Norwich Research Park (University of East Anglia, John Innes Centre, Quadram Institute and Earlham Institute). Through a rotation‐based programme, EDESIA PhD students will train in a wide range of disciplines across the translational pathway of nutrition research, including analyses of epidemiological datasets, assessment of nutritional bioactives, biochemical, genetic, cell biological and functional analyses of plant metabolites, in vitro analyses in tissue and cell cultures, investigation of efficacy in animal models of disease, investigation of effects on composition and functioning of the microbiota and human intervention studies. Research rotations add a breadth of knowledge, outside of the main PhD project, which benefits the students and can be brought into project design. This comprehensive PhD training programme will allow the translation of science into guidelines for healthy eating and the production of nutritionally improved food crops, leading to innovative food products, particularly for prevention and treatment of chronic diseases where age is a major risk factor. In this article, we summarise the programme and showcase the experiences of the first cohort of students as they start their substantive PhD projects after a year of research rotations.

## INTRODUCTION

In an era where preventive medicine is becoming increasingly important due to an ageing population and the rapidly increasing prevalence of obesity, optimised diets are key to improving health (Collaborators, 2019; Ezzati & Riboli, 2013). Nutrition and reduction in risk of ill‐health are currently centre stage globally, and the launch of the EAT‐Lancet commission report (Willett et al., 2019) highlighted that food represents one of the greatest health and environmental challenges of the twenty‐first century and stressed the urgent need to increase the consumption of plant‐rich diets. The report describes the immense challenge facing humanity to provide the growing world population with healthy, plant‐based diets from sustainable food systems. Three different statistical approaches were used to quantify the health and environmental impacts of adopting a planetary diet, characterised by high plant‐based foods and limited animal food/unhealthy foods, calculating that such a change would prevent 19%–24% adult deaths and overall approximately 11 million deaths per year.

The NHS Long Term Plan (2019) also has prevention at its heart, with the largest economic burden on the NHS predicted to stem from poor diets and food‐related ill health costing £5.8 billion per year (Scarborough et al., 2011). The NHS Five Year Forward View (2014) set out its ambition for prevention of non‐communicable disease and a radical upgrade in prevention and public health strategies.

**FIGURE 1:**
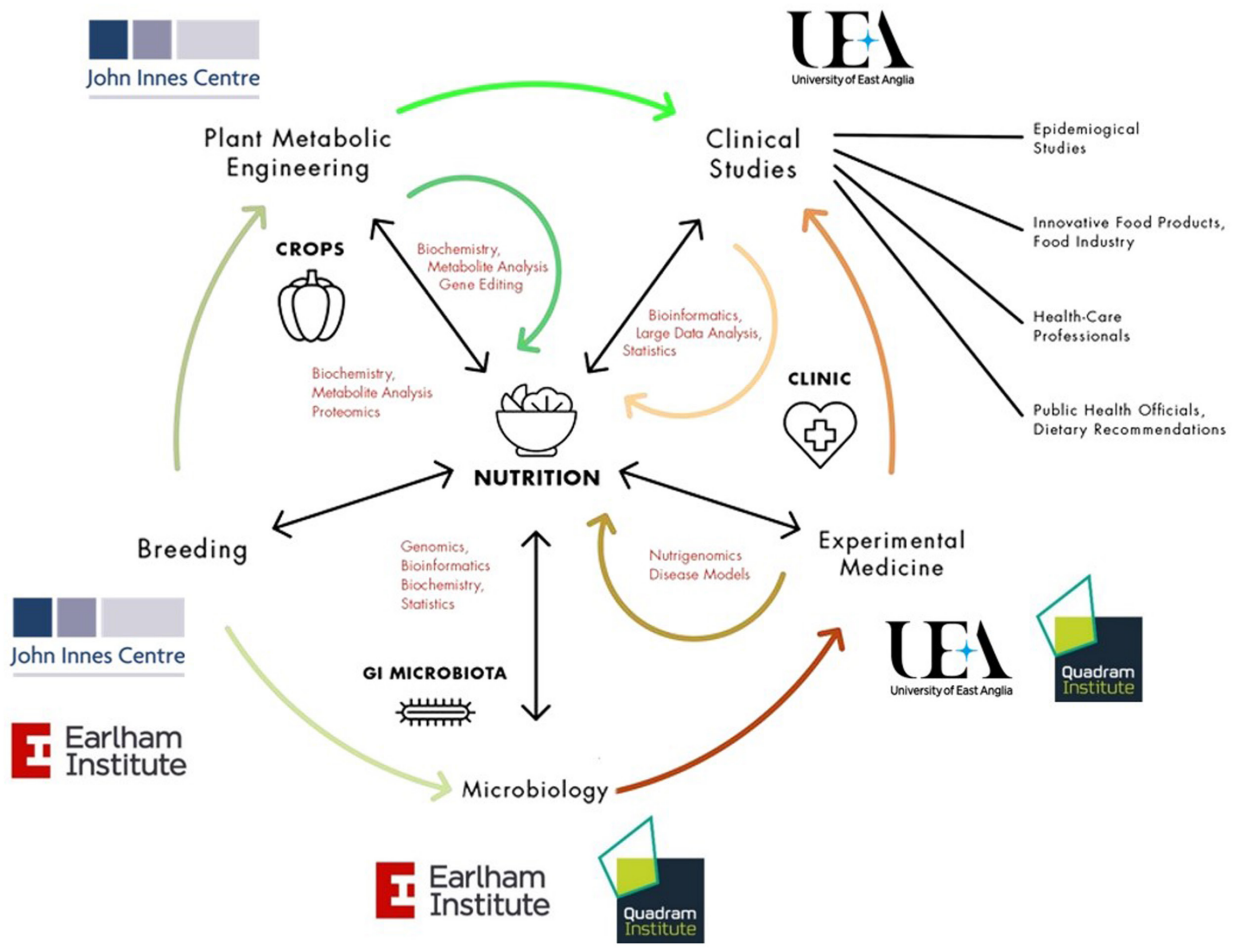
Multidisciplinary research strengths in nutrition across the Norwich Research Park

The unique EDESIA PhD programme is focused on major aspects of plant‐based nutrition and health, from crop to clinic, drawing on the world‐class interdisciplinary research expertise across the Norwich Research Park (NRP) (plant science, mechanistic understanding of nutrition, clinical trials, population‐based studies, public health; Figure 1). The NRP, which includes three BBSRC funded research institutes (the John Innes Centre, Quadram Institute Bioscience and the Earlham Institute), UEA and the Norfolk & Norwich University Hospital is, to our knowledge, the largest European Centre with a predominant research focus on Plants, Diet and Health. Together, we are providing a step change in multi‐disciplinary research training for PhD students at the intersection of nutrition and health research, and addressing the key issues highlighted by the MRC Review of Nutrition and Human Health Research (2017). This includes unravelling the complex inter‐relationship between plant‐based foods, metabolism, the gut microbiota and health outcomes.

Many plant‐sourced, essential vitamins and minerals, as well as phytonutrients, are associated with reduced risk of obesity and related chronic diseases such as cancers (prostate, breast and colon cancers, in particular), inflammatory diseases like inflammatory bowel disease, metabolic diseases such as type 2 diabetes and cardiovascular disease (Collaborators, 2019; Ezzati & Riboli, 2013). Perhaps because they are non‐essential, or because their beneficial effects have been recognised only recently, the role of bioactive dietary compounds from plants in promoting health has been largely overlooked in reviews of nutrition, including the MRC Review of Nutrition and Human Health Research (2017), despite a huge body of evidence and authoritative reviews on the beneficial effects of fruit and vegetables and wholegrains in the diet on protecting against chronic disease (Collaborators, 2019; Ezzati & Riboli, 2013).

More than half of contemporary public health problems could be prevented through dietary change (Collaborators, 2019, Ezzati & Riboli, 2013). Key to improved health are plant‐based foods which supply most essential micronutrients as well as fibre, resistant starch, polyphenols, flavonoids and carotenoids in human diets. In the most recent Global Burden of Disease Report (Collaborators, 2019), a low fruit intake was a leading dietary risk factor, calculated to be responsible for 2 million deaths and 65 million ‘disability adjusted life years’, worldwide. The loss of plant‐based, unrefined foods from human diets means that ever more people are burdened with nutritional insecurity and associated chronic diseases, fuelled by the global obesity epidemic (Willett et al., 2019).

Understanding how plant‐based foods promote and protect health will underpin effective dietary recommendations, food formulations, food choices, food products, agriculture and the health of future societies, worldwide, bringing with it improved quality of life. This also has potential for significant environmental impacts. This programme addresses several United Nations Sustainable Development Goals (2021).

Overall, the EDESIA PhD programme aims to provide the required training to address the shifting food security challenge of the twenty‐first century: increasing access to a nutrient‐rich diet, rather than a calorie‐rich one.

## PHD PROGRAMME

**FIGURE 2:**
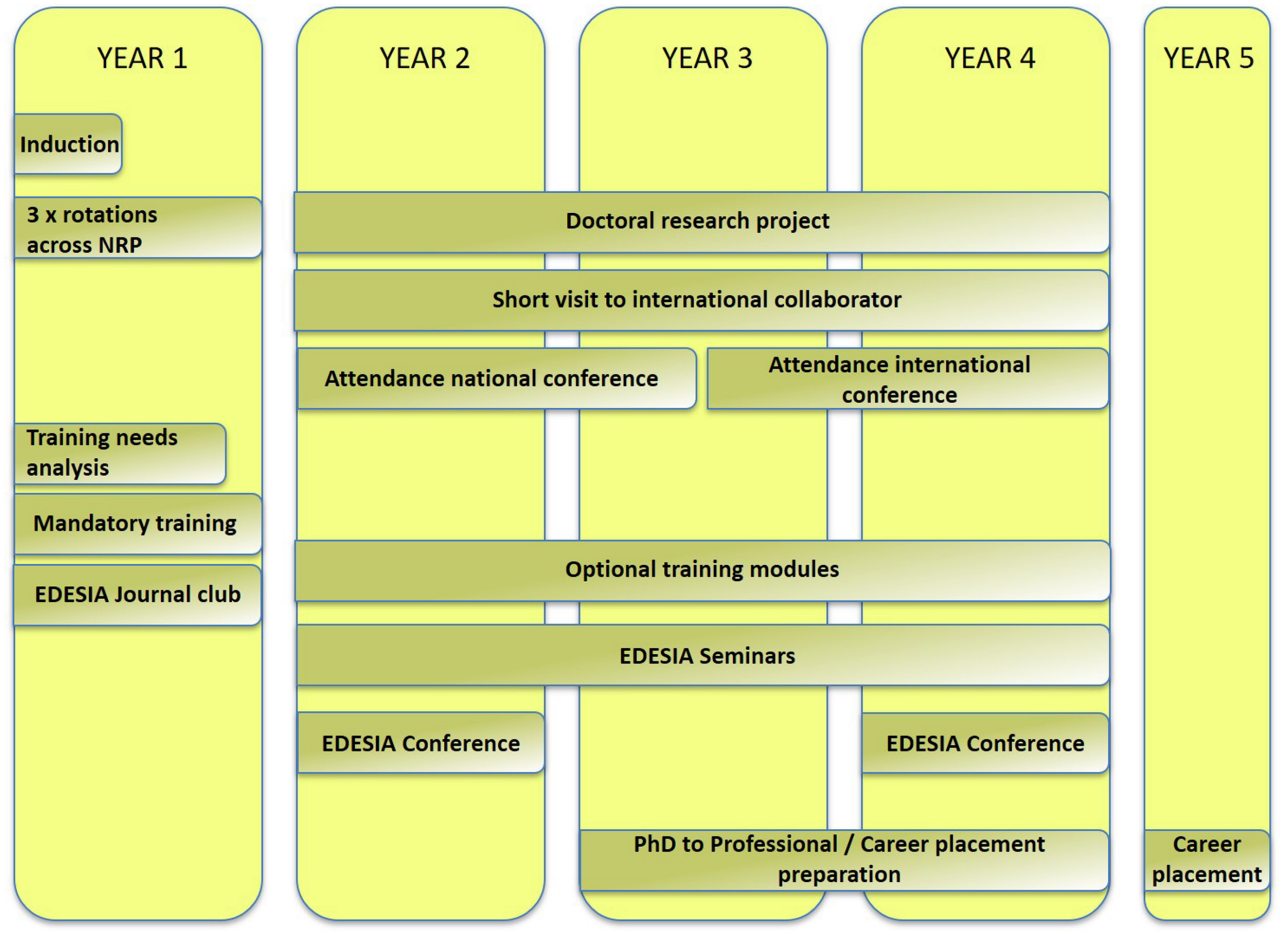
Structure of EDESIA: Plants, Food and Health: a cross‐disciplinary PhD programme from crop to clinic. NRP, Norwich Research Park

The EDESIA PhD programme is rotation based (see Figure 2). In an initial rotation year, EDESIA students select three research rotations across NRP, giving them the opportunity to train in a wide range of disciplines across the translational pathway of nutrition research. This includes the development and testing of nutritional bioactive compounds, biochemical, genetic, cell biological and functional analysis of plant metabolites, in vitro analysis of the biological activity of key metabolites in model tissues and cell cultures, investigation of mechanisms of action in animal models of disease, human intervention studies and complex statistical analyses of large prospective cohort datasets working with the best phenotyped cohorts, worldwide. They will have access to metabolic engineering of plants which has enabled the development of crops containing enhanced or optimised concentrations of specific nutrients, including state‐of‐the‐art approaches to speed breeding and genome editing of crops (Martin, 2018; Martin et al., 2011; Martin et al., 2013; Martin & Li, 2017; Ramirez‐Gonzalez et al., 2018). They can explore the function of the gut microbiome in the absorption of plant‐based foods and their further metabolism by human tissues, combined with the use of the latest ‘omics’ technologies to measure changes in metabolism and gene expression to establish the sites of action of particular food‐derived metabolites and the mechanisms by which they confer health benefits (Curtis et al., 2019; Davidson et al., 2018). This is complemented by expertise in bioinformatics, mathematical biology and metagenomics. They can understand the mechanisms of action of plant‐based foods and their metabolites by using numerous in vitro and in vivo models of human physiology and disease (Cassidy et al., 2013; Curtis et al., 2019; Davidson et al., 2013; Davidson et al., 2018). This can lead into opportunities to learn and develop novel modelling approaches in nutrition intervention trials.

Hypotheses are also often generated from the analyses of prospective and cross‐sectional population‐based studies, which enable optimisation in the design of randomised controlled trials. For nutrition, the best available evidence integrates the totality of evidence (Blumberg et al., 2010; FDA, 2022) including epidemiological, clinical trial and mechanistic studies to inform and refine public health advice to reduce the risk of chronic disease. This comprehensive PhD training programme aims to allow the translation of science into refined guidelines for healthy eating and the production of nutritionally improved crop‐based foods.

**FIGURE 3:**
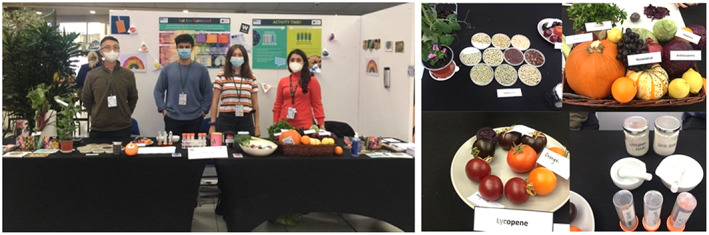
Norwich Science Festival 2021—EDESIA students

The EDESIA programme is also supported by a variety of cohort activities (Figure 3). In the rotation year, there are a number of specific training modules, as well as a journal club enabling students to explore their research areas. Throughout the programme, there is a seminar series where speakers are invited to NRP and knowledge exchange and networking are enabled. A student‐led biennial EDESIA Conference adds to this and enables students to gain knowledge in the organisation of meetings. The Wellcome Trust PhD programmes are also unique in offering a funded work placement following submission of the PhD thesis.

The EDESIA programme integrates elements of the Wellcome Trust's mission to ‘reimagine research’ and enhance research culture. Five students are being recruited each year, with fee waiver (from UEA) for two international students to make the programme as inclusive as possible. Support for applications is also provided via virtual ‘question and answer’ sessions with the programme directors, as well as detailed FAQ online. UEA has also strongly engaged in postgraduate student mental health via the *COURAGE* project, the results of which have helped inform practices at UEA and nationally. Alongside funding for the PhD programmes, the Wellcome Trust have awarded funding to UEA for Biomedical Vacation Scholarships. These specifically engender widening participation, giving students from appropriate backgrounds an opportunity to engage in research and explore opportunities within the PhD programmes and beyond.

## EXPERIENCES, RESEARCH ROTATIONS AND PHD PROJECTS OF THE FIRST COHORT OF STUDENTS

### Mark Bal—Lars Østergaard laboratory, John Innes Centre

A key facet of the EDESIA programme is to use the research rotations to move outside your comfort zone. One of my rotations centred on the evaluation of a class of seaweed cell wall metabolites known as fucoidans as a possible dietary intervention for sufferers of osteoarthritis (OA). Fucoidans are polysulfated polysaccharides which were found in an in vitro study to concentrate an endogenous inhibitor of cartilage breakdown (TIMP3) in the extracellular medium of a cartilage cell line (Troeberg et al., 2009). Furthermore, fucoidans were also found to be capable of binding directly to and inhibiting cartilage‐degrading enzymes (matrix metalloproteinases, MMPs) in kinetic assays (Rose & Kooyman, 2016). I had not worked on human biology before, so this rotation gave me a newfound knowledge and appreciation of medical science, which now allows me to participate in diverse fields ranging from clinical research through to my original focus of plant science.

My full PhD project uses the knowledge of nutritional science and digestion that I accrued over another earlier rotation, working on wheat, to explore a possible link between the developmental biology of legumes and their potential health‐promoting metabolites (e.g. resistant starch structures, amino acid profiles). While already widely appreciated for their high levels of fibre and plant protein, as well as several micronutrients (Rebello et al., 2014), the staggering morphological diversity of cultivated peas (Ellis et al., 2021) remains untapped in the context of health. Since the developmental processes that underlie pea fruit shape and maturation are hypothesised to affect photosynthesis and plant‐to‐seed resource allocation (and thus the nutritional profiles of the resultant food products), my project aims to search for the theoretical, health‐promoting pod ‘ideotype’ in pea and other legumes. Ranging in experimental approach from pea genetics and molecular biology through to food biochemistry and models of digestibility (Brodkorb et al., 2019), this transdisciplinary research topic will play a role in further establishing the intersection between agricultural and nutritional science as a widespread field of study.

### Yvie Morgan—Janneke Balk laboratory, John Innes Centre

Iron deficiency anaemia (IDA) is the most prevalent nutrient deficiency worldwide (Pasricha et al., 2021). As trends in plant‐based diets continue to increase, dietary reliance upon plants as a source of iron will be of greater importance for sufficient iron intake. My interest in IDA dietary alleviation is what drew me to my current PhD project. During my first rotation in the UEA Norwich Medical School, I used nutritional epidemiology to analyse the UK national diet and nutrition survey, exploring potential associations between micronutrient status and COVID‐19 risk. Whilst conducting my second rotation at the Quadram Institute Bioscience, I learnt how to effectively simulate in vitro digestion of plant‐based foods and measure micronutrient release by elemental analyses. My third rotation in the Balk laboratory at the John Innes Centre led to my main PhD project within this lab and this utilises knowledge from the previous rotations. Owing to limited natural variation in iron content of the wheat grain, a genetic modification approach has been developed in the Baulk laboratory to biofortify the grain with iron. Ectopic expression of a single gene encoding a wheat iron transporter resulted in redistribution of iron to the starchy endosperm, resulting in 2‐3‐fold increases in iron in the white flour (Connorton et al., 2017). I will utilise 2 years of field‐grown high‐iron wheat, to explore iron bioavailability (release and absorption) in baked products. I will compare biofortified bread rolls with the chemically fortified bread with respect to iron bioavailability, using an in vitro Caco‐2 cell culture model. This material may be used in future human clinical trials to understand the potential of the high‐iron bread to alleviate IDA.

### Alicia Nicklin, Stephen Robinson laboratory, Quadram institute

Around 55 200 new cases of breast cancer are diagnosed in the UK every year, making it the UK's most common cancer (Cancer Research UK, 2021). To combat this disease, it is vital to develop novel therapeutic and preventative strategies. Emerging research has shown that the gut microbiota plays a pivotal role in breast cancer initiation, pathogenesis as well as treatment efficacy (Goedert et al., 2015; Laborda‐Illanes et al., 2020). As such, modulating the gut microbiota—to restore and promote gut homeostasis—is an attractive, non‐invasive therapeutic avenue. My research PhD will employ multidisciplinary skills to assess mechanisms underpinning the gut‐tumour axis and provide dietary intervention strategies, such as potential probiotic strains and prebiotics to modulate the gut microbiome. Skills, attained through 10‐week rotation projects, have been topical and indispensable for my current and future work, including metabolomics skills which I am now harnessing to identify potential gut microbiota‐derived anti‐tumorigenic metabolites. In addition, the EDESIA rotation PhD scheme has promoted cross‐institute collaborations for my project—harnessing the plant science expertise at John Innes Centre, as well as expertise in microbiome research and pre‐clinical studies at the Quadram Institute. Overall, the overarching aim for my PhD project is to define a cancer‐protective microbiome so that therapeutic strategies involving probiotics and prebiotics can be employed, most‐likely supplementing existing cancer treatments.

### Yang Yue—Cathie Martin laboratory, John Innes Centre

As a part of the first EDESIA cohort, I undertook three rotation projects in the UEA Norwich Medical School, the Quadram Institute and the John Innes Centre. In addition, I have attended a constellation of training programmes on topics that included food business market value chains, human nutrition, and hardcore bioinformatics. The journal club was one of the most rewarding experiences for me, as well as the rotation review meetings. The rotation scheme was invaluable for me, where I learned new experimental and communication skills which helped me to identify my strengths and limitations. Most importantly, the rotation experience enabled me to select with confidence the topic for my full PhD project.

**FIGURE 4:**
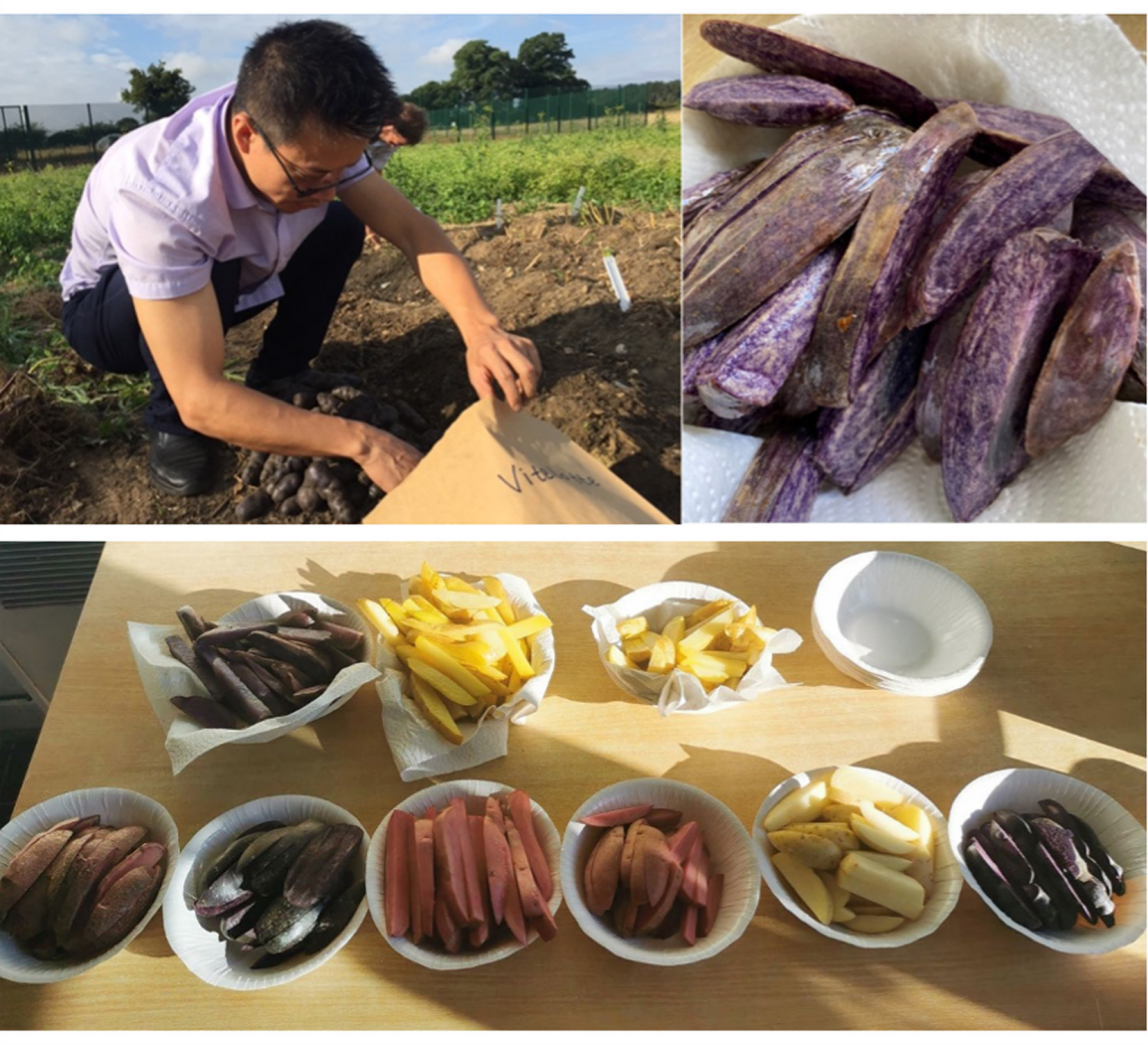
Research can be fun—potato harvesting and chip tasting (Yang Yue)

My third rotation project (as well as my full PhD project) aimed to investigate how the consumption of coloured potatoes containing anthocyanins might contribute to gut and brain health. My long‐term objective is to gain in‐depth insights into the mechanisms by which anthocyanin‐enriched foods benefit health, especially their efficacy in protecting against chronic disease and/or in enhancing mood and memory. During my rotation, I found that coloured potatoes show different responses to Rapid Visco Analysis (RVA) compared to white potatoes. RVA provides an indirect measurement of starch viscosity that mimics the effects of cooking on starch. I am keen to investigate whether anthocyanins can behave like proanthocyanidins (PAs) to improve the resistance of starch. PAs can be added to maize starch, to increase its resistance to digestibility and to help control postprandial hyperglycaemia for type 2 diabetic patients. I had so much fun making different coloured potato chips at the JIC with the support from the lab and designing a questionnaire about their taste. My full PhD project is built on extensive collaborations with experts from the John Innes Centre, Norwich Medical School and the Quadram Institute. I hope my research will enable me to provide evidence‐based recommendations for diets to help relieve the socioeconomic burden of unhealthy ageing (Figure 4).

### Aryana Zardkoohi—Nathalie Juge laboratory, Quadram institute

My PhD project is at the interface between plant sciences, gut microbiome and human health. Before selecting the main project, I experienced three rotations, where in each I was able to acquire different sets of skills. In the first rotation, I learnt programming and applied statistics by analysing data from the Osteoarthritis Initiative (Osteoarthritis Initiative, 2022); my second rotation was based the University of East Anglia, where I was able to study the effects of plant‐derived compounds on a polyacrylamide hydrogel model to study the effects of these compounds and matrix stiffness on vascular smooth muscle cells (Minaisah et al., 2016). Finally, during my third rotation, based at the Quadram Institute Bioscience, in the group where I am now conducting my PhD project, I studied the in vitro gut‐barrier function effects of resistant starch from peas, in a collaborative project with Imperial College London. Altogether, the rotation year gave me experimental skills and technical knowledge that have helped me towards the main PhD project, as well as invaluable interdisciplinary collaboration across the fields of medicine, nutrition, plant science and cell biology.

Currently, I am working with *Pisum sativum*, also known as garden peas, focusing on the role of raffinose family oligosaccharides (RFOs) produced by these plants on human nutrition and health. RFOs have been associated with the protection from desiccation in plants (Sengupta et al., 2015) but when consumed by humans in legumes, they can also lead to abdominal discomfort and flatulence, and have even been considered as anti‐nutrients (Glencross et al., 2003; Leske et al., 1995; Reddy et al., 1984). Nonetheless, there is evidence that members of the human gut microbiota such as *Ruminococcus gnavus* can utilise RFOs through several α‐galactosidases (Cervera‐Tison et al., 2012; Ejby et al., 2016; Lafond et al., 2020; Zartl et al., 2018).

In my project, I will be generating pea mutants with reduced RFO content from mutagenesis approaches at the John Innes Centre and studying the effect of these mutants on gut health at the Quadram Institute Bioscience. Experimentally, I will use in vitro models of digestion and fermentation, and gut barrier function with intestinal organoids derived from human donors from the Norfolk & Norwich University Hospital endoscopy unit.

Collectively, these interdisciplinary approaches aim to explore the partial or complete reduction of RFOs from legumes to increase human consumption, focusing on the impacts these compounds have on human gut health.

## CONCLUSION

Optimised diets are key to healthy ageing and the prevention of disease. The EDESIA, Plants, Food and Health PhD programme builds capacity in this area, training the next generation of nutrition researchers.

The ability to undertake three research rotations across diverse areas of nutrition research adds a breadth to the EDESIA PhD programme that would not be possible in traditional PhD training. This comes through in the experiences of the first cohort of students as they begin their substantive projects, equipped with a wide knowledge of additional research areas that will benefit their research.

Being part of a group of students undertaking research in nutrition, with cohort training and activities, seminars and conference organisation, adds to the research culture and helps to provide an additional level of mutual support and knowledge exchange.

We look forward to the outcomes of the EDESIA PhD programme as it progresses.

## CONFLICT OF INTEREST

The authors have no conflict of interests to declare.

## Data Availability

Data sharing not applicable ‐ no new data generated, or the article describes entirely theoretical research.
